# Editorial: Advances in neonatal-perinatal palliative care

**DOI:** 10.3389/fped.2023.1307584

**Published:** 2023-11-06

**Authors:** Steven R. Leuthner, DonnaMaria E. Cortezzo

**Affiliations:** ^1^ Division of Neonatology, Children’s Wisconsin, Milwaukee, WI, United States; ^2^ Division of Critical Care and Palliative Care, Children’s Wisconsin, Milwaukee, WI, United States; ^3^ Department of Pediatrics, Medical College of Wisconsin, Milwaukee, WI, United States; ^4^ Center for Bioethics and Medical Humanities, Medical College of Wisconsin, Milwaukee, WI, United States; ^5^ Division of Neonatal and Pulmonary Biology, Cincinnati Children’s Hospital Medical Center, Cincinnati, OH, United States; ^6^ Division of Pain and Palliative Medicine, Cincinnati Children’s Hospital Medical Center, Cincinnati, OH, United States; ^7^ Department of Pediatrics, University of Cincinnati College of Medicine, Cincinnati, OH, United States; ^8^ Department of Anesthesiology, University of Cincinnati College of Medicine, Cincinnati, OH, United States; ^9^ Division of Neonatology, Connecticut Children’s Medical Center, Hartford, CT, United States; ^10^ Division of Pain and Palliative Care, Connecticut Children’s Medical Center, Hartford, CT, United States; ^11^ Fetal Care Program, Connecticut Children’s Medical Center, Hartford, CT, United States; ^12^ Department of Pediatrics, University of Connecticut School of Medicine, Farmington, CT, United States

**Keywords:** perinatal palliative care, neonatal palliative care, life-limiting diagnosis, neonatal, uncertain prognosis

**Editorial on the Research Topic** Advances in neonatal-perinatal palliative care

Neonatal-Perinatal Palliative Care is a complete, multidisciplinary approach to care that integrates Obstetrics, Neonatology, and Palliative Medicine with the goal of providing total care for families when there is a potentially life-limiting or medically complex fetal or neonatal diagnosis (1). Technological advances have led to earlier and more frequent fetal diagnoses, allowing families time to navigate complex medical decisions (2, 3). They have also led to more treatment options, care paths, and, at times, uncertainty. Neonatal-perinatal palliative care provides psychosocial and spiritual support throughout the family's journey, helping them navigate goals of care and complex medical decisions, with a focus on comfort and quality of life, sometimes concurrently with life-prolonging interventions (4, 5). The multidisciplinary approach allows for collaboration and cohesive care across multiple subspecialties involved and phases of care.

With recognition of the importance of neonatal-perinatal palliative care for families and providers, the field has grown significantly in recent years (6–10). There are several hundred perinatal palliative care programs and an increased focus on research. Recent research highlights benefits of neonatal-perinatal palliative care, experiences with life-limited diagnoses, trends in medical care, and barriers (7, 11–13). Despite these recent clinical and academic advances, there remains a paucity of data, practice variation, and a lack of expert voice in many areas within the field. These areas include: education and program development, ethics in the context of the changing medico-legal climate, sub-specialty collaboration for medically complex neonates, advanced symptom management for infants with chronic conditions and at the end of life, the impact of and counseling around fetal interventions or within fetal care centers, and disparities.

In this gathering of 7 peer-reviewed manuscripts from experts in their respective fields, we aimed to fill some of the gaps in the literature. The collection includes one article on virtual training in perinatal palliative care, two articles sharing single center experiences, two articles discussing perinatal palliative care through a cultural lens, and two articles providing examples of the expansion of perinatal palliative care beyond the lethal defect and concurrently with life-prolonging medical choices.

In “Evaluation of learning transfer after perinatal/neonatal palliative care virtual training course,” Brady et al. share their descriptive prospective study evaluating a virtual training course developed during the pandemic. This serves as a valuable educational resource with great potential for expanding comfort in providing perinatal palliative care services. It highlights the potential utility of alternate platforms for training providers.

The articles that share their single center experience include a description of guidelines based on a wealth of experience, and a study evaluating a centers perception of the end-of-life care with the implementation of guidelines. In “Perinatal Palliative Care: Focus on Comfort,” McCarthy et al. provide a follow up to a previous article published in Frontiers that described the program at Columbia University Irving Medical Center. Through this Perspective piece, the authors share their guidelines and walk providers through practical details for care. They describe tools and strategies they found necessary in assessing and providing comfort care over their 15-year time frame to aid in program development. In “Clinician's perception of care at the end of life in a quaternary NICU,” Lauren Imai et al. aimed to assess clinician perception on end-of-life care in a NICU by utilizing the Pediatric Intensive Care Unit-Quality of dying and Death survey, as well as a global rating of clinician satisfaction. It was conducted over 3 Epochs-before, during, and after the implementation of end-of-life guidelines. The study revealed that while most responses were high some reported major challenges and room for improvement in certain areas. Symptom management, communication, and satisfaction in education scores improved over Epochs. Put together, these articles demonstrate that guidelines might support improvements in the delivery of neonatal comfort and end-of-life care.

With expansion of the concepts of palliative care, it is important to recognize cultural circumstances that may be new to the western trained physicians. In “Muslim perspectives on palliative care in perinatal and neonatal patients: a mini-review” Shoaib et al. provide a review of three clinical scenarios in which withdrawing or withholding of life-sustaining medical interventions are discussed from the Muslim religious and scholarly perspective. This article offers cultural insight into how these ethical questions are addressed in this growing community. While every family is an individual, recognizing where an individual's values might be rooted in has great benefit. This more global understanding of perinatal palliative care is further expanded in “Perinatal palliative care in sub-Saharan Africa: recommendations for practice, future research and guideline development,” by Abayneh et al. The authors explore differences between high vs. low- and middle-income countries in how perinatal palliative care is provided. They discuss difficulties in communication, resources, and lack of research and understanding of cultural differences.

The final two articles support the idea that perinatal palliative care is moving beyond the role of only caring for the fetus that will imminently die. In, “Role of palliative care in fetal neurological consultations: Guiding through uncertainty and hope,” Cortezzo et al. share the value of palliative care involvement in prenatal diagnoses of uncertainty. Providers can be a consistent team to help support families perhaps through comfort care at birth, but also through longer survivorship within the NCIU or beyond. In the same vein, the “Role of palliative care in centers performing maternal-fetal interventions,” by Rholl et al. expands the concept that perinatal palliative care should be provided concurrently with aggressive fetal interventions. Maternal fetal decision-making is complex and palliative care providers have skills in helping families navigate difficult decisions in this arena. As these interventions come with risks and variable outcomes, a team that follows through with support in decision-making has value.

As the field of neonatal-perinatal palliative care continues to grow and evolve there remains a need to perform robust research on best practices and educate the communities that these services can support. With innovative therapies, families are tasked with navigating more complex decisions and the role of neonatal-perinatal palliative care continues to expand. We hope readers find these articles add some pieces to this enlarging puzzle of neonatal-perinatal palliative care.
